# Human amniotic epithelial cell-derived exosomes promote conjunctival goblet cells proliferation and mucin secretion

**DOI:** 10.1097/MD.0000000000049647

**Published:** 2026-07-17

**Authors:** Ting Meng, Shuiping Yang, Wenjia Wu, Miao Gong, Yanming Zhang

**Affiliations:** a Department of Ophthalmology, Shenzhen People’s Hospital (The First Affiliated Hospital, Southern University of Science and Technology; The Second Clinical Medical College, Jinan University), Shenzhen, Guangdong, China; b School of Medical Technology and Nursing, Shenzhen Polytechnic University, Shenzhen, Guangdong, China; c Engineering Research Center of Advanced Glasses Manufacturing Technology, Ministry of Education, Donghua University, Shanghai, China.

**Keywords:** conjunctival goblet cells, exosome, human amniotic epithelial cells, mucin secretion, proliferation

## Abstract

To evaluate the influence of human amniotic epithelial cell-derived exosomes (hAECs-Exo) on the proliferation, apoptosis, and cell cycle of conjunctival goblet cells in vitro. Exosomes were isolated from hAECs via ultracentrifugation and characterized using transmission electron microscopy and nanoparticle tracking analysis software. The presence of exosomal marker proteins CD63 and CD81 was confirmed through western blot. Conjunctival goblet cells were identified by morphology and immunofluorescence staining for cytokeratin 7 and mucin 5AC (MUC5AC). Subsequently, the conjunctival goblet cells were treated with hAECs-Exo or phosphate-buffered saline (control). MUC5AC expression and secretion were quantified by enzyme-linked immunosorbent assay and western blot. Cell viability, apoptosis, and cell cycle distribution were assessed using Cell Counting Kit-8 assays, Annexin V/propidium iodide staining, and flow cytometry, respectively. hAECs-Exo vesicles demonstrate positive expression for CD63 and CD81 markers. In contrast, conjunctival goblet cells exhibit fluorescent signals indicative of cytokeratin 7 and MUC5AC expression. hAECs-Exo were found to enhance the expression and secretion of MUC5AC in conjunctival goblet cells. In addition, treatment with hAECs-Exo resulted in a notable increase in the viability of conjunctival goblet cells and a reduction in apoptosis compared to treatment with phosphate-buffered saline. Following exposure to hAECs-Exo, there was a significant decrease in the proportion of G1 and G2 phase cells in conjunctival goblet cells, accompanied by a notable increase in the proportion of S-phase cells. The application of hAECs-Exo has been shown to enhance the viability of goblet cells within the conjunctival tissue and sustain their physiological activity.

## 1. Introduction

The conjunctiva, covering approximately two-thirds of the ocular surface, plays a vital role in corneal integrity and tear film stability.^[1]^ Its epithelium contains goblet cells that secrete mucins, which lubricate the ocular surface and support antimicrobial defense.^[2,3]^ Globally, ocular diseases affect 3.54% of people aged 40 to 80, with dry eye syndrome reaching 11.3% in those over 50.^[4,5]^ While preservative-containing eye drops are commonly used, agents such as benzalkonium chloride can damage conjunctival tissue and induce inflammation.^[6]^ In addition, conjunctival injury may result from chemical or thermal burns, Stevens–Johnson syndrome, infections, ocular cicatricial pemphigoid, or surgical procedures, often leading to goblet cell dysfunction.^[7]^ Such damage significantly impairs vision and quality of life, underscoring the need to protect conjunctival health during ocular treatment. In particular, the loss or dysfunction of conjunctival goblet cells is a central pathological feature in several of these conditions, including dry eye disease, chemical burns, ocular cicatricial pemphigoid, and postsurgical conjunctival injury. Restoring goblet cell number and function, therefore, represents a promising therapeutic target for such disorders.

At present, there is a limited number of successful therapies available for conditions linked to conjunctival tissue impairment. Over the past few years, procedures such as autologous or allogeneic limbal transplantation and ex vivo cultured epithelial transplantation have been employed in the management of various diseases affecting conjunctival tissue, notably limbal epithelial stem cell deficiency.^[8]^ In the last 20 years, human amniotic epithelial cells (hAECs) have garnered significant attention in the field of regenerative medicine due to their various advantageous characteristics.^[9]^ These include a low frequency of mutations, minimal immunogenicity and tumorigenicity, abundant availability, lack of ethical concerns, and successful transplantation in animal models for treating a range of conditions such as liver, cardiac, ophthalmological, ovarian, musculoskeletal, and neurological diseases.^[10]^ Transplanted hAECs have demonstrated enhancements in wound healing and tissue repair, as well as exhibiting antifibrotic and anti-inflammatory properties.^[11,12]^ Noteworthy is the observation from certain studies indicating the renal protective effects of hAEC stem cell therapy in diverse kidney diseases. Despite the limited differentiation of hAECs into adequate cell numbers for organ reconstruction, their paracrine function is deemed crucial, prompting a significant focus on investigating the paracrine role of hAECs in research.^[13]^ It is interesting to note that nearly all stem cells, including hAECs, possess the ability to secrete exosomes. Exosomes are a type of extracellular vesicle characterized by a phospholipid bilayer and ranging in size from 30 to 200 nm. These vesicles are capable of transporting various signaling molecules, such as lipids, proteins, mRNA, microRNAs, and noncoding RNAs, to recipient cells, thereby influencing cellular function and metabolic processes.^[14,15]^

Recent research has indicated that hAECs can impact apoptosis and M2 polarization in hypoxia-reoxygenation-damaged HK-2 cells and macrophages through the release of exosomes.^[16]^ While the reparative effects of hAECs-derived exosomes (hAECs-Exo) on recipient cells have been observed, their impact on conjunctival goblet cells remains unexplored. Specifically, existing studies have primarily focused on the effects of intact amniotic membrane or amniotic epithelial cells on conjunctival epithelial cell differentiation, progenitor cell expansion, or transdifferentiation into corneal phenotypes.^[17,18]^ More recent work has investigated extracellular vesicles from hAECs for dry eye disease treatment, but with emphasis on corneal epithelial repair and overall ocular surface inflammation.^[19]^ In contrast, our study is distinct in that it directly investigates the impact of isolated hAECs-Exo on the specific biological functions of purified human conjunctival goblet cells in vitro, namely their proliferation, apoptosis, cell cycle, and mucin secretion. This cell-type-specific and mechanistic focus has not been previously reported. Consequently, this study aims to investigate the effects of hAECs-Exo on the proliferation, apoptosis, and cell cycle of conjunctival goblet cells in vitro, with the goal of establishing a foundational understanding for potential therapeutic strategies targeting conjunctival damage diseases, particularly those characterized by goblet cell deficiency or dysfunction.

## 2. Materials and methods

### 2.1. Source of specimens and ethical considerations

hAECs and human conjunctival tissues were both obtained through approved ethical procedures at Shenzhen People’s Hospital. hAECs were isolated from amniotic tissue samples collected from 3 healthy mothers undergoing cesarean section. Human conjunctival tissues were obtained as surgical waste from 3 donors undergoing eye surgeries unrelated to limbal pathology, specifically strabismus correction or pterygium excision. The tissue collection process adhered to the principles outlined in the Declaration of Helsinki and was approved by the Internal Review Committee of Subjects at Shenzhen People’s Hospital (approval number: 2024-308-01). Informed consent was obtained from all participants involved in the study.

### 2.2. Isolation and identification of hAECs

#### 2.2.1. Isolation of hAECs

The fresh amniotic tissue was thoroughly rinsed with sterile phosphate-buffered saline (PBS), then digested with trypsin-ethylenediaminetetraacetic acid on the tissue, filtered through a cell sieve, and centrifuged to obtain a single-cell suspension. The obtained cell precipitate was cultured in Dulbecco modified Eagle medium containing 5.5 mmol/L glucose, 10% fetal bovine serum, and 2% penicillin/streptomycin at 37°C and 5% CO_2_.

#### 2.2.2. Identification of hAECs

The identity of hAECs was confirmed by their characteristic cobblestone-like epithelial morphology under phase-contrast microscopy. Further verification was performed using flow cytometry, which demonstrated positive expression of typical epithelial and stem cell markers (CD29, CD166, CD90) and negative expression of hematopoietic markers (e.g., CD45).^[20]^ hAECs from the third passage were used for subsequent experiments.

### 2.3. Isolation and characterization of hAECs-Exo

#### 2.3.1. Isolation of exosomes

To isolate exosomes secreted by hAECs, a seeding of 1 × 10^6^ hAECs was performed in a 25 cm^2^ cell culture dish. Upon reaching 80% cell fusion, the medium was replaced with exosome-free medium (41210ES50, Yeasen). Following 48 hours of culture, the cell culture medium was harvested. Subsequently, the collected medium was centrifuged at 12,000 rpm for 10 minutes at 4°C to obtain the cell supernatant. The supernatant was then transferred to an ultracentrifuge (Optima XPN, Beckman) and centrifuged at 120,000 g for 2 hours. The resulting supernatant was discarded, and the pellet was collected, resuspended in prechilled PBS, and the protein concentration was determined using a BCA kit (P0012, Beyotime). The resuspended pellet was then aliquoted and stored at −80°C for future use.

#### 2.3.2. Transmission electron microscopy for morphological assessment

In order to observe the shape of the isolated exosomes, the exosomes were first mixed with 2.5% glutaraldehyde, fixed overnight at 4°C, resuspended with PBS, centrifuged at 120,000 g for 2 hours, then washed once again with PBS, the exosomes were added dropwise to the formvar-carbon-coated grid, negatively stained with phosphotungstic acid aqueous solution for 60 seconds, and exosome formation was observed under 80 kV conditions using a transmission electron microscope (Hitachi, Japan, H7500 TEM) and photographed for recording.

#### 2.3.3. Nanoparticle tracking analysis for size distribution

In order to assess the particle size distribution and concentration of exosomes, the dimensions of the vesicles were analyzed through dynamic light scattering with the utilization of a ZetasizerNano ZS90 analysis system from Malvern Instruments. Subsequently, a particle size distribution map was generated, depicting the estimated particle size (nm) on the x-axis and the corresponding particle count on the y-axis.

#### 2.3.4. Western blot for exosomal marker identification

Western blotting analysis was performed to confirm the presence of exosome marker proteins. A total of 1 mL of RIPA lysis buffer (BL504A, Biosharp) was used to lyse hAECs-Exo, and then the mixture was centrifuged at 12,000 rpm for 10 minutes at 4°C. The supernatant was collected and the protein concentration was determined according to the guidelines provided by the BCA kit (P0012, Beyotime). Then, 20 μg of total protein was mixed with sodium dodecyl sulfate (SDS) protein loading buffer (G2527, Lablad) to completely denature it. The total protein was then separated by 10% SDS-polyacrylamide gel electrophoresis and transferred to a polyvinylidene fluoride membrane (LabSelect). The polyvinylidene fluoride membrane was treated with protein-free rapid blocking solution (PS108P, EpiZyme) for 15 minutes, and then incubated with primary antibodies against exosome markers CD63 (1:1000, ABclonal, A19023) and CD81 (1:1000, ABclonal, A4863), as well as the negative marker Grp94 (1:1000, ABclonal, A0989) overnight. The membrane was incubated with HRP-conjugated secondary antibody (AS014, ABclonal, 1:15000) for 2 hours. The bands were treated with ECL (C05-07004, Bioss) solution and then developed using the WB imaging system (JP-K300, Jiapeng). The images were analyzed using ImageJ software to confirm the successful isolation of exosomes.

### 2.4. Isolation and identification of human conjunctival goblet cells

#### 2.4.1. Isolation of conjunctival goblet cells

For the isolation of conjunctival goblet cells, the collected conjunctival tissue fragments were washed with PBS, minced into small fragments, and cultured in RPMI-1640 medium supplemented with 10% fetal bovine serum, 2 mM glutamine, and 1% penicillin/streptomycin under standard conditions (37°C, 5% CO_2_). Fibroblasts were removed by differential adhesion, and primary goblet cells were obtained through explant culture. After cell outgrowth, residual tissue and non-goblet cells were removed based on morphological characteristics. Only cells within the third passage were used for experiments to minimize phenotype alteration from extended passaging.

#### 2.4.2. Identification of conjunctival goblet cells via immunofluorescence

1 × 10^6^ conjunctival goblet cells were inoculated into a 6-well cell culture plate. After the cells completely adhered, the cell culture medium was removed and the cells were rinsed 3 times with precooled PBS. Then, the cells were fixed with 4% paraformaldehyde for 30 minutes, and then washed twice with PBS. Next, the cells were treated with PBS solution containing 0.2% Triton-X for 30 minutes, washed after that, and then added with PBS solution containing 1% BSA for further treatment for 30 minutes. The cells were incubated with the primary antibodies against cytokeratin 7 (CK-7, anti-mouse, 1:500, ab9021, Abcam) and against mucin 5AC (MUC5AC, anti-mouse, 1:500, ab3649, Abcam) overnight. After washing, the cells were incubated with the secondary antibody mouse IgG H&L Alexa Fluor® 488 (1:1000, excitation wavelength 495 nm, emission wavelength 519 nm, ab150113, Abcam) for 1 hour, then washed and stained with 4′,6-diamidino-2-phenylindole for 10 minutes. The expression of CK-7 and MUC5AC was used to confirm the identity and purity of the goblet cells. The negative control group did not use the primary antibodies.

### 2.5. Experimental design and treatment of conjunctival goblet cells

Conjunctival goblet cells were seeded at a uniform density in culture plates appropriate for each subsequent assay. For all experiments, after seeding, the cell culture wells/plates were randomly assigned to either the hAECs-Exo treatment group or the PBS control group using a computer-generated random number table. Group allocation was performed prior to treatment, and all subsequent analyses were conducted using the assigned group labels. The treatment group received hAECs-Exo at a final concentration of 100 µg/mL. The control group received an equal volume of sterile PBS. Following a 48 hours incubation period under standard culture conditions (37°C, 5% CO_2_), cells and supernatants were harvested for analysis.

### 2.6. Assessment of exosome uptake by conjunctival goblet cells

1,1′-dioctadecyl-3,3,3′,3′-tetramethylindocarbocyanine perchlorate (Dil) is a long-chain di-alkyl carboxylate dye with a unique structure that can bind to lipid-soluble biological structures, thus having high lipophilicity.^[21]^ In this study, it was used for exosome labeling for uptake research. To investigate the uptake of exosomes by conjunctival goblet cells, the cells were assigned according to the predefined randomization scheme described above. A total of 100 µg/mL of exosomes were incubated with 2 µM of Dil solution (40718ES50, Yeasen) at 37°C for 5 minutes. Subsequently, the exosomes labeled with Dil were added to the cells of the experimental group. To eliminate the influence of nonspecific dye absorption, the control group received the same amount of Dil solution in PBS (without exosomes). After coculture for 48 hours, the cell culture medium was removed and the cells were rinsed 3 times with precooled PBS. Then, the cells were fixed with 4% paraformaldehyde for 30 minutes, washed with PBS, and observed using a fluorescence microscope (DM IL LED, Leica) for the Dil signal within the cells (emission wavelength: 553 nm, excitation wavelength: 570 nm).

### 2.7. Assessment of hAECs-Exo on goblet cell proliferation and survival

#### 2.7.1. Cell viability assay (Cell Counting Kit-8)

The impact of hAECs-Exo on the viability of conjunctival goblet cells was assessed using the Cell Counting Kit-8 (CCK-8) assay kit (C0038, Beyotime). Conjunctival goblet cells were seeded in 96-well plates at a concentration of 5 × 10^4^ cells/mL in 100 μL of medium. The plates were then incubated in a cell culture incubator until cell adhesion was complete. Cells were then randomly assigned to 2 groups: the experimental group (hAECs-Exo group), which received 10 μL of hAECs-Exo at a concentration of 100 μg/mL, and the control group, which received an equal volume of PBS. Following a 48-hour incubation period, 10 μL of CCK-8 solution was introduced to each well. After a 1-hour incubation, the optical density at 450 nm was measured using an iMark microplate reader (Bio-Rad) to determine cell viability. Viability was calculated relative to the control group (set as 100%).

#### 2.7.2. Apoptosis assay (Annexin V/propidium iodide staining)

To assess the impact of hAECs-Exo on cell survival during conjunctival repair, we evaluated its effects on apoptosis in conjunctival goblet cells. The effect of hAECs-Exo on the apoptosis level of conjunctival goblet cells was evaluated using the Annexin V Alexa Fluor 647/propidium iodide (PI) apoptosis detection kit (catalog number 40304ES20, Yeasen). Conjunctival goblet cells were cultured at a concentration of 8 × 10^5^ cells/mL in 6-well plates. Upon achieving complete adherence of the cells, 10 μL of hAECs-Exo (100 μg/mL) was introduced to incubate with the conjunctival goblet cells for a period of 48 hours. Subsequently, the cells were harvested, centrifuged at 300 g for 5 minutes at 37°C, washed twice with PBS, and the cell concentration was adjusted to 1 × 10^6^ cells/mL. The cells were then suspended in 100 μL of PBS, followed by the addition of 5 μL of Annexin V/Alexa Fluor 647 and 10 μL of 20 μg/mL PI solution to the PBS. The mixture was thoroughly combined and allowed to incubate for 15 minutes at room temperature in the absence of light. The fluorescence intensities of FITC and PI (Ex.488 nm and Em.530 nm) in the cells were subsequently evaluated using flow cytometry (CytoFLEX S, Beckman).

#### 2.7.3. Cell cycle analysis (PI staining)

To evaluate the effect of hAECs-Exo on cell proliferation during conjunctival repair, we examined its influence on the cell cycle progression of conjunctival goblet cells. The effect of hAECs-Exo on the cell cycle of conjunctival goblet cells was evaluated using the cell cycle staining kit (40301ES50, Yeasen). Conjunctival goblet cells were seeded in 6-well culture plates at a density of 8 × 10^5^ cells/mL (the same density used in the apoptosis assay) and incubated for 24 hours. Subsequently, 10 μL of hAECs-Exo (100 μg/mL) was added to the conjunctival goblet cells, followed by a 48-hour incubation period. The cells were then harvested into centrifuge tubes, centrifuged at 300 g for 5 minutes at 37°C, washed twice with PBS, and the cell concentration was adjusted to 5 × 10^5^ cells/mL. The cells were resuspended in 300 μL of PBS, centrifuged again, and 100 μL of RNase A reagent was added to resuspend the cells. The cell suspension was then incubated in a 37°C water bath for 30 minutes, followed by the addition of 400 μL of PI reagent (50 μg/mL), which was thoroughly mixed and incubated at a low temperature in the dark for 30 minutes. The PI signal intensity was quantified using flow cytometry (CytoFLEX S, Beckman) with excitation at 488 nm and emission at 530 nm.

### 2.8. Assessment of hAECs-Exo on MUC5AC expression and secretion

#### 2.8.1. MUC5AC secretion (enzyme-linked immunosorbent assay)

The MUC5AC enzyme-linked immunosorbent assay detection kit (D711278) was used to assess the effect of hAECs-Exo on the secretion of MUC5AC by conjunctival goblet cells. Conjunctival goblet cells were cultured at a concentration of 8 × 10^5^ cells/mL in 6-well plates. Once the cells had adhered completely, 10 μL of hAECs-Exo (100 μg/mL) were added to the cells for a 48-hour incubation period. Following this, the cell culture medium was collected and centrifuged at 12,000 g for 5 minutes at 4°C to obtain the medium supernatant. Subsequently, enzyme-linked immunosorbent assay detection was carried out according to the provided instructions, and the absorbance at 450 nm was measured using an iMark microplate reader (Bio-Rad). A regression curve was generated based on the absorbance values of the standard, enabling the calculation of the MUC5AC content within the cells.

#### 2.8.2. MUC5AC protein expression (western blot)

After the treatment, the conjunctival goblet cell lysate was prepared. The protein concentration was determined according to the experimental procedure described in the previous section 2.3.4 “Western blot for exosomal marker identification,” and the total protein was separated by SDS-polyacrylamide gel electrophoresis. The membrane was incubated with the primary antibody MUC5AC (anti-Rabbit, 1:1000, ABclonal, A17325) and β-actin antibody (anti-Rabbit, 1:5000, ABclonal, AC026) overnight. The next day, the sample was incubated with the HRP-labeled secondary antibody (Goat anti-Rabbit IgG [H + L], 1:10000, AS014) for 2 hours. The bands were treated with ECL solution, and the band intensity was quantified using ImageJ software, and the expression of MUC5AC was normalized to β-actin.

### 2.9. Data analysis

All data are presented as mean ± standard deviation from at least 3 independent experiments. Statistical analyses were performed using GraphPad Prism 9.0 software. Comparisons between the 2 study groups – the hAECs-Exo treatment group and the PBS control group – were conducted using an unpaired two-tailed Student *t* test. A *P* value < .05 was considered statistically significant.

## 3. Results

### 3.1. Identification and characterization of hAECs-Exo

**Figure 1.**
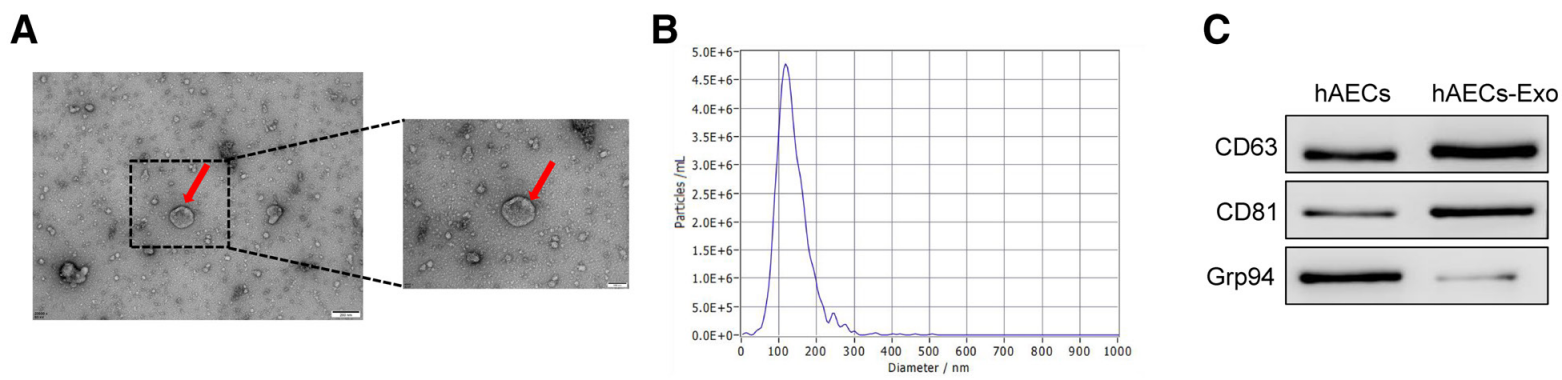
Identification and characterization of hAECs-Exo. (A) Morphology of exosomes in the hAECs-Exo group was observed under transmission electron microscopy (Scalar bar = 200 nm). (B) Diameter of exosomes in the hAECs-Exo group was analysis under NTA. (C) Western blot was used to observe the expression of CD63, CD81, and Grp94 proteins in hAECs-Exo. hAECs-Exo = human amniotic epithelial cell-derived exosomes, NTA = nanoparticle tracking analysis.

Examination via transmission electron microscopy revealed the characteristic oval-shaped vesicle morphology of hAECs-Exo (Fig. 1A). Analysis of nanoparticle size distribution demonstrated that the exosomes within the hAECs-Exo group exhibited diameters ranging from 45 to 130 nm (Fig. 1B). Furthermore, western blot analysis confirmed the presence of exosomal marker proteins CD63 and CD81 in hAECs-Exo, while the expression level of Grp94 was notably low (Fig. 1C). These findings collectively affirm the successful acquisition of hAECs-Exo.

### 3.2. Identification of human conjunctival goblet cells

**Figure 2.**
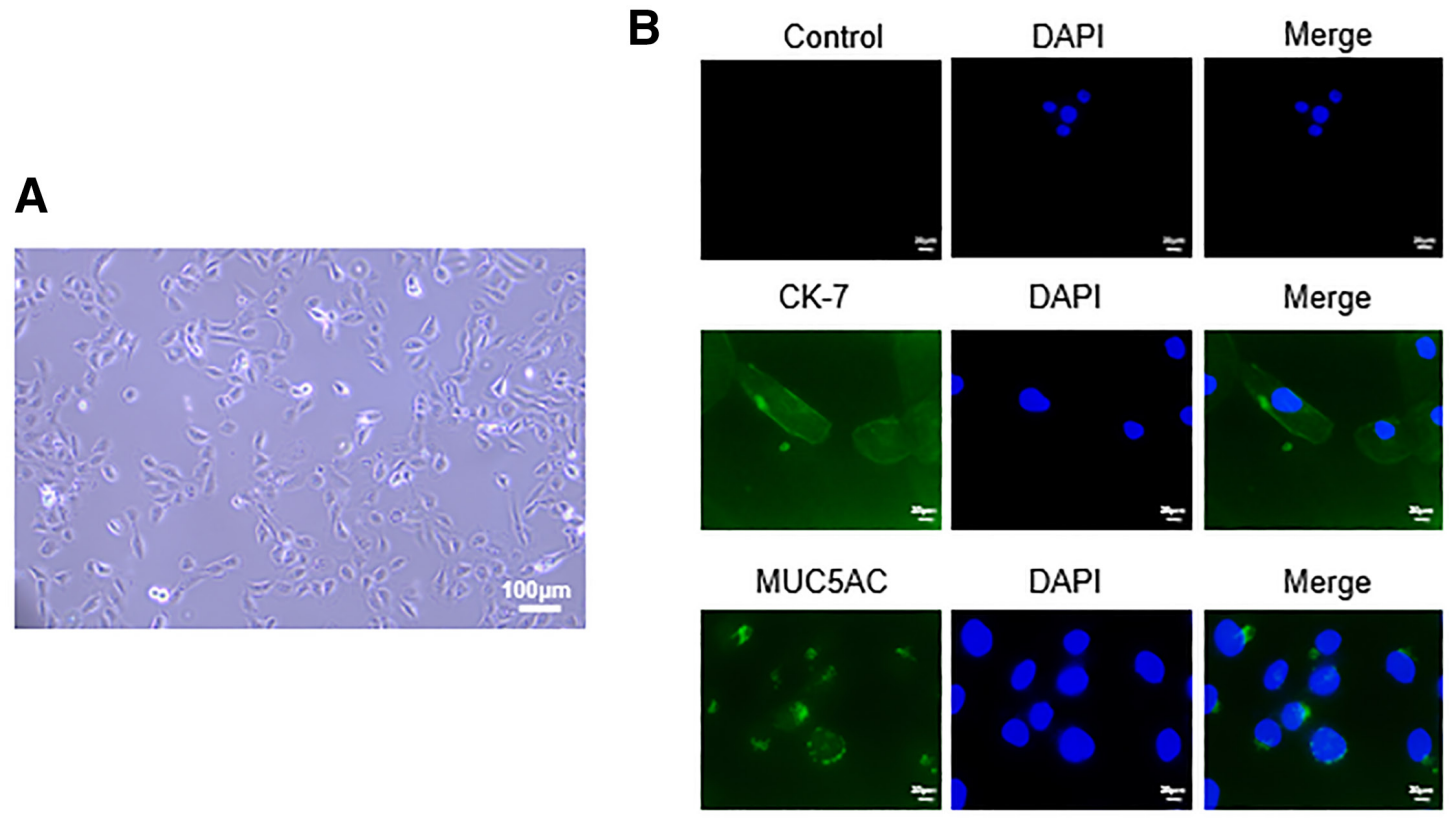
Identification of human conjunctival goblet cells. (A) The morphological structure of isolated human conjunctival goblet cells was observed under microscopic (Scalar bar = 100 μm). (B) Immunofluorescence was used to detect the fluorescence levels of CK-7 and MUC5AC in human conjunctival goblet cells (Scalar bar = 20 μm). CK-7 = cytokeratin 7, DAPI = 4′,6-diamidino-2-phenylindole, MUC5AC = mucin 5AC.

To enhance the circumstances, the initial step involved the isolation of conjunctival goblet cells from donated conjunctival tissue. Subsequently, the morphology of the isolated conjunctival goblet cells was examined using a microscope, which showed a characteristic cobblestone-like epithelial appearance with well-defined polygonal cells and distinct intercellular borders (Fig. 2A), followed by the assessment of CK-7 and MUC5AC expression in these cells through cellular immunofluorescence. The expression of CK-7 and MUC5AC in the conjunctival goblet cells was then visualized using fluorescence microscopy (Fig. 2B).

### 3.3. Uptake of hAECs-Exo and effects on the viability of human conjunctival goblet cells

**Figure 3.**
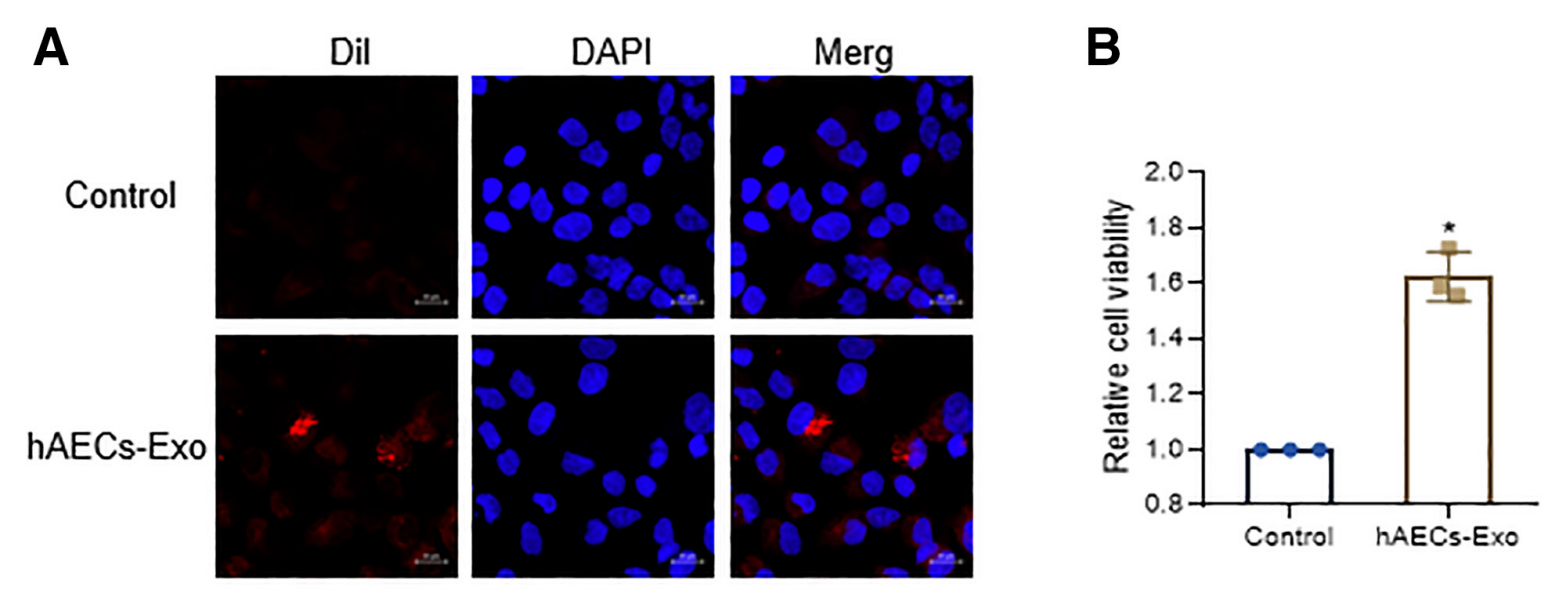
Uptake of hAECs-Exo and effects on the viability of human conjunctival goblet cells. (A) The uptake of Dil-labeled hAECs-Exo by conjunctival goblet cells was observed. (B) CCK-8 was used to detect the viability of cells in the control group and hAECs-Exo group. *P < .05 versus control group. CCK-8 = Cell Counting Kit-8, Dil = 1,1′-dioctadecyl-3,3,3′,3′-tetramethylindocarbocyanine perchlorate, hAECs-Exo = human amniotic epithelial cell-derived exosomes.

After labeling hAECs-Exo by Dil, a large amount of Dil signal can be visualized microscopically in the conjunctival goblet cells (Fig. 3A). Subsequently, the CCK-8 kit was used to detect changes in the activity of conjunctival goblet cells, and the results showed that hAECs-Exo could significantly increase the viability of conjunctival goblet cells compared to the control group (*P* < .05; Fig. 3B). It can be seen that hAECs-Exo can promote the activity of conjunctival goblet cells.

### 3.4. hAECs-Exo inhibits apoptosis and regulates the cell cycle of human conjunctival goblet cells

**Figure 4.**
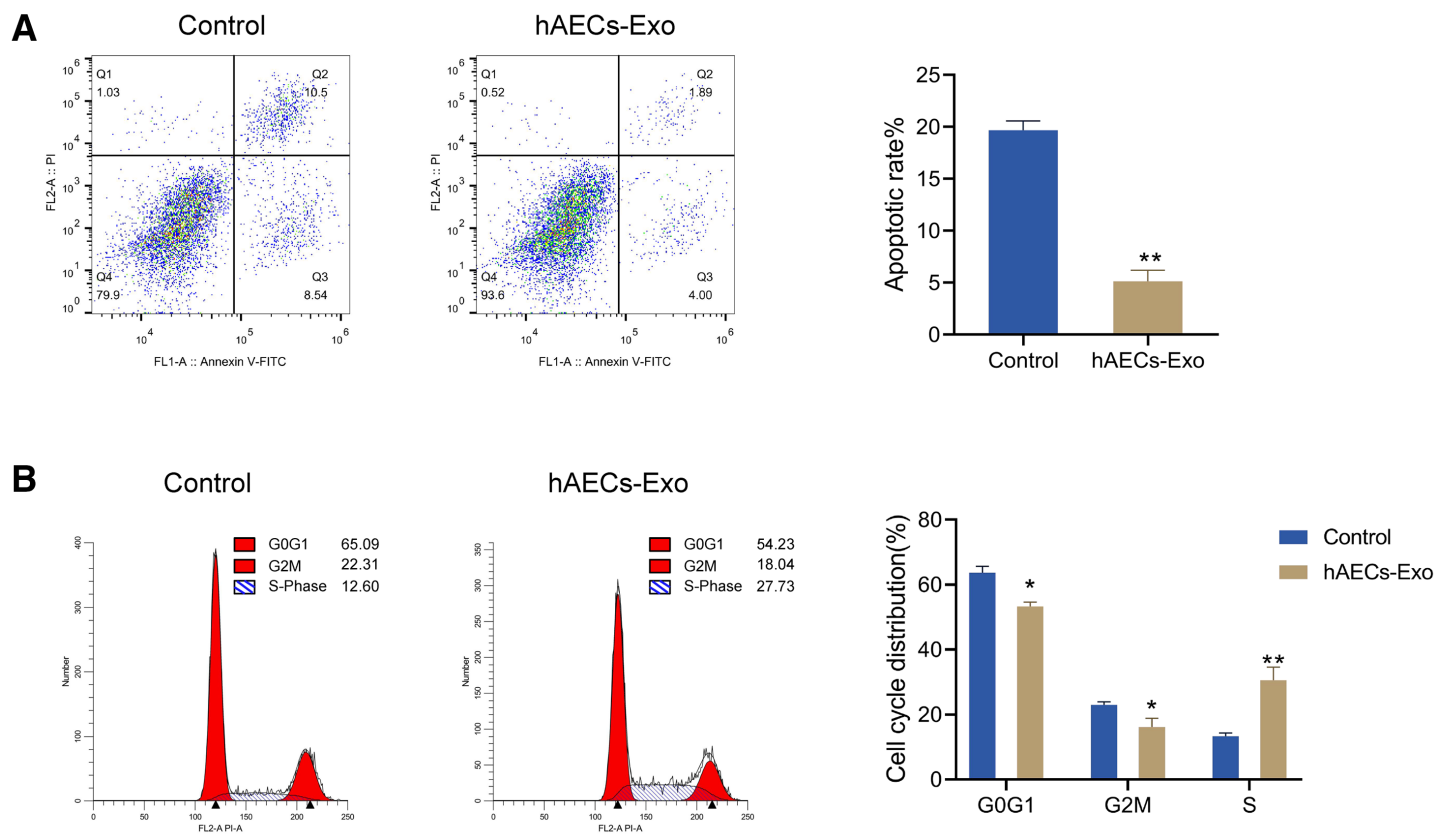
hAECs-Exo inhibits apoptosis of human conjunctival goblet cells and regulates the cell cycle. (A) Annexin V Alexa Fluor 647/PI staining was used to observe the apoptosis level of cells in the control group and hAECs-Exo group. (B) PI staining was used to observe the cell cycle of cells in the control group and hAECs-Exo group. *P < .05, **P < .01 versus control group. hAECs-Exo = human amniotic epithelial cell-derived exosomes, PI = propidium iodide.

First, Annexin V Alexa Fluor 647/PI staining showed that hAECs-Exo significantly reduced the level of apoptosis relative to the control group (*P* < .01; Fig. 4A). At the same time, according to PI staining, 65.09% of the cells in the control group were in G0G1 phase, 22.31% were G2M, and 12.60% were in S phase, while the proportion of G0G1 and G2 phase cells in the conjunctival goblet cell cycle incubated with hAECs-Exo was significantly reduced, 54.23% and 18.04%, respectively, and the proportion of S-phase cells (27.73%) was significantly increased (Fig. 4B).

### 3.5. hAECs-Exo promotes the expression and secretion of MUC5AC in human conjunctival goblet cells

**Figure 5.**
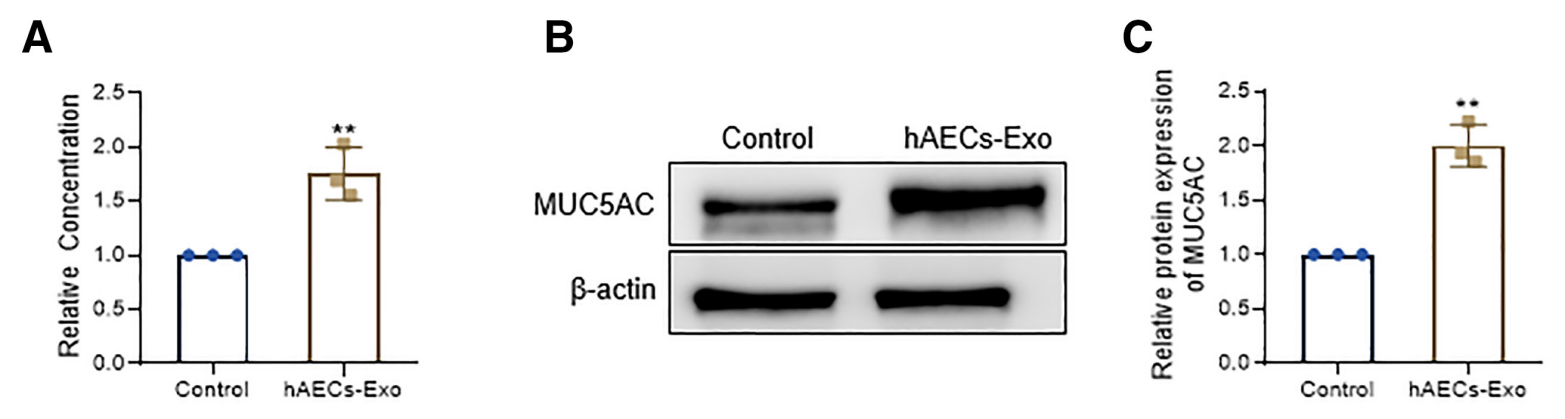
hAECs-Exo promotes the expression and secretion of MUC5AC in human conjunctival goblet cells. (A) The level of MUC5AC in the supernatant of cell culture in the control group and hAECs-Exo group was detected by ELISA. (B and C) Western blot was used to detect the protein level of MUC5AC in the control group and hAECs-Exo group. **P < .01 versus control group. ELISA = enzyme-linked immunosorbent assay, hAECs-Exo = human amniotic epithelial cell-derived exosomes, MUC5AC = mucin 5AC.

The findings revealed a notable increase in the levels of MUC5AC secreted by conjunctival goblet cells following incubation with hAECs-Exo in comparison to the control group (*P* < .01; Fig. 5A). In addition, the protein expression of MUC5AC in conjunctival goblet cells was assessed through western blot analysis, which indicated a significant upregulation in MUC5AC expression in cells treated with hAECs-Exo relative to the control group (*P* < .01; Fig. 5B, C). These outcomes suggest that hAECs-Exo plays a role in enhancing the expression and secretion of MUC5AC in human conjunctival goblet cells.

## 4. Discussion

This study aimed to investigate the effects of exosomes derived from hAECs-Exo on the function and survival of human conjunctival goblet cells in vitro. The findings reveal that hAECs-Exo significantly enhanced the viability of conjunctival goblet cells while reducing their apoptosis and promoting cell cycle progression, as indicated by an increased proportion of S-phase cells. Furthermore, hAECs-Exo upregulated both the expression and secretion of the key mucin MUC5AC. Notably, these functional effects were mediated by exosomes that were successfully isolated from hAECs and efficiently internalized by the target goblet cells. Collectively, these results suggest that hAECs-Exo holds promise as a cell-free therapeutic agent for supporting conjunctival goblet cell health and function.

Firstly, we successfully isolated hAECs-Exo with the size ranging from 45 to 130 nm and positive expression of exosomal markers CD63 and CD81. Although the size range observed in this study is not consistent with the previously reported size ranges of hAECs-Exo (50–150 nm or 41–82 nm),^[16,22]^ these differences may be attributed to donor-specific factors such as gestational age and individual metabolism.^[23]^ It is notable that the functional activity of exosomes is not directly related to their particle size.^[24]^ We confirmed through various characterization methods that exosomes were successfully isolated from primary hAECs, highlighting the reliability of this model system for subsequent functional assays. This rigorous characterization laid a crucial foundation for attributing the observed biological effects specifically to exosomes.

Secondly, we found that hAECs-Exo significantly upregulated the intracellular protein expression and extracellular secretion of MUC5AC in conjunctival goblet cells. MUC5AC is the main gel-forming mucin secreted by conjunctival goblet cells and is crucial for tear film stability and ocular surface protection.^[2,25]^ Previous studies have shown that cytokines such as interleukin-13 or signaling pathways related to epithelial cell differentiation can stimulate the production of MUC5AC in goblet cells.^[2,26]^ However, there is a lack of evidence linking stem cell-derived exosomes to mucin regulation in goblet cells. This study identified hAECs-Exo as a novel cell-free paracrine medium that can enhance mucin secretion, filling this gap. This effect is consistent with the role of amniotic membrane transplantation in promoting goblet cell proliferation and conjunctival health, such as in the treatment of ocular cicatricial pemphigoid.^[27]^ hAECs-Exo can be isolated, purified, and used as an in-cell therapeutic approach, serving as a supplement or alternative strategy to traditional amniotic membrane transplantation for ocular surface repair.^[28,29]^ This discovery has highlighted the potential application value of hAECs-Exo in restoring tear film integrity in conjunctival diseases.

Thirdly, we found that hAECs-Exo significantly promoted the survival and proliferation of conjunctival goblet cells, increasing the survival rate by approximately 80%. At the same time, it inhibited their apoptosis, reducing the apoptosis rate by about 15%; and it also promoted the cell cycle progression, with the proportion of S-phase cells significantly increasing from 12.60% to 27.73%. These research results indicate that the functional advantages of hAECs can be effectively transmitted through their exosomes, in a way that collaboratively inhibits cell apoptosis and stimulates proliferative activity, thereby promoting the survival and functional recovery of conjunctival goblet cells. This pro-survival and pro-proliferative effect is consistent with the regenerative functions of hAECs-Exo in other tissues. For example, hAECs-Exo has been shown to enhance fibroblast migration and proliferation in skin wound healing^[14,30]^ and protect renal tubular epithelial cells from cisplatin-induced cell death.^[31]^ The increase in S-phase cells indicates that hAECs-Exo may stimulate the cell cycle progression. Kang et al proposed based on RNA sequencing data that hAECs-Exo may regulate cell cycle and DNA repair-related genes (such as Ccnb1, Cdk1),^[31]^ and other studies have shown that the tumor necrosis factor-alpha/mitogen-activated protein kinase and phosphatidylinositol 3-kinase/protein kinase B/mammalian target of rapamycin pathways play a role in hAECs-Exo-mediated repair.^[32,33]^ Our research provides new evidence that the extensive cell protection and pro-proliferative properties of hAECs-Exo are particularly targeted at the often overlooked but clinically crucial conjunctival goblet cells, providing a targeted strategy to combat the loss of goblet cells in ocular surface diseases.

Several methodological strengths of this study enhance the credibility and validity of our findings. First, hAECs were derived from the innermost layer of the placenta closest to the fetus and obtained from amniotic membranes after delivery, without any invasive procedures for cell harvesting. This ethically favorable and noninvasive source minimizes donor-related bias and supports the translational potential of hAEC-based therapies. Second, we successfully isolated extracellular vesicles actively secreted by hAECs and rigorously confirmed their exosomal identity through the positive expression of the canonical membrane markers CD63 and CD81, together with their characteristic size and morphology. This multiparametric validation ensures the purity and reliability of the isolated exosome population. Importantly, our study specifically focused on exosomes secreted by viable hAECs prior to any decellularization procedures. Since decellularization eliminates all cellular components, including extracellular vesicles, this approach allowed us to investigate the genuine paracrine effects mediated by hAECs-Exo, rather than residual matrix-associated factors. Together, these methodological considerations strengthen the interpretability of our results and support the biological relevance of hAECs-Exo in regulating conjunctival goblet cell function.

Although this study has many advantages, we must acknowledge its limitations. Firstly, this study employed an in vitro experimental method, and the complex in vivo microenvironment of the ocular surface may affect the efficacy of hAECs-Exo. The in vivo efficacy still needs to be verified. Secondly, the molecular mechanism behind the observed effects has not been fully elucidated. Future research should explore specific signaling pathways related to goblet cell regulation and the substances carried by exosomes. Moreover, the current study lacks detailed donor information (such as the mother’s age, health status, and ocular diagnosis). In comparative studies on hAECs-Exo or conjunctival goblet cell biology, donor data usually contain more detailed information, which may affect the biological characteristics of cells and exosomes. In the previous studies, the amniotic epithelial cell donors and conjunctival tissue donors reported that the mothers were free from infectious diseases or systemic disorders (such as AIDS, hepatitis),^[34]^ and the subjects were without symptoms or signs of tear dysfunction.^[35]^ In future research, we will expand the sample size and conduct a comprehensive analysis of donors to improve reproducibility and classify the therapeutic effects of exosomes. Finally, we have not conducted a systematic analysis of individual differences in human amniotic cells and exosome components. Future research should establish in vivo models such as rabbit dry eye models^[36]^ to verify the therapeutic effect of local application of hAECs-Exo. In addition, analyzing clinical samples after amniotic transplantation can help verify the distribution of exosomes in the body and their contribution to the clinical outcome of amniotic transplantation.

## 5. Conclusion

The results above demonstrate that exosomes derived from hAECs-Exo have a significant impact on enhancing the viability of conjunctival goblet cells, suppressing apoptosis, and supporting cell survival through cell cycle regulation. Furthermore, hAECs-Exo have the ability to enhance the expression and secretion of MUC5AC in conjunctival goblet cells, thereby preserving the functionality of these cells. In summary, our research indicates the potential therapeutic value of hAECs-Exo in treating conditions related to conjunctival tissue damage and offers a theoretical basis for the application of hAECs transplantation.

## Author contributions

**Conceptualization:** Ting Meng, Shuiping Yang, Wenjia Wu, Miao Gong, Yanming Zhang.

**Data curation:** Ting Meng, Shuiping Yang, Miao Gong, Yanming Zhang.

**Formal analysis:** Ting Meng, Shuiping Yang, Wenjia Wu, Miao Gong, Yanming Zhang.

**Funding acquisition:** Ting Meng, Shuiping Yang, Miao Gong, Yanming Zhang.

**Investigation:** Ting Meng, Shuiping Yang, Wenjia Wu, Miao Gong, Yanming Zhang.

**Methodology:** Ting Meng, Shuiping Yang, Miao Gong, Yanming Zhang.

**Project administration:** Ting Meng, Shuiping Yang, Wenjia Wu, Miao Gong, Yanming Zhang.

**Resources:** Ting Meng, Shuiping Yang, Miao Gong, Yanming Zhang.

**Software:** Ting Meng, Shuiping Yang, Wenjia Wu, Miao Gong, Yanming Zhang.

**Supervision:** Ting Meng, Shuiping Yang, Miao Gong, Yanming Zhang.

**Validation:** Ting Meng, Shuiping Yang, Wenjia Wu, Miao Gong, Yanming Zhang.

**Visualization:** Ting Meng, Shuiping Yang, Miao Gong, Yanming Zhang.

**Writing – original draft:** Ting Meng, Shuiping Yang, Wenjia Wu, Miao Gong, Yanming Zhang.

**Writing – review & editing:** Ting Meng, Shuiping Yang, Miao Gong, Yanming Zhang.
